# Ubiquitin Ligase Parkin Regulates the Stability of SARS-CoV-2 Main Protease and Suppresses Viral Replication

**DOI:** 10.1021/acsinfecdis.3c00418

**Published:** 2024-02-22

**Authors:** Li Zhou, Ruochuan Liu, Heather Pathak, Xiaoyu Wang, Geon H. Jeong, Pratima Kumari, Mukesh Kumar, Jun Yin

**Affiliations:** † Department of Chemistry and Center for Diagnostics and Therapeutics, Georgia State University, Atlanta, Georgia 30303, United States; ‡ Department of Biology and Center for Diagnostics and Therapeutics, Georgia State University, Atlanta, Georgia 30303, United States

**Keywords:** E3 ubiquitin ligase, Parkin, SARS-CoV-2, main protease, mitophagy

## Abstract

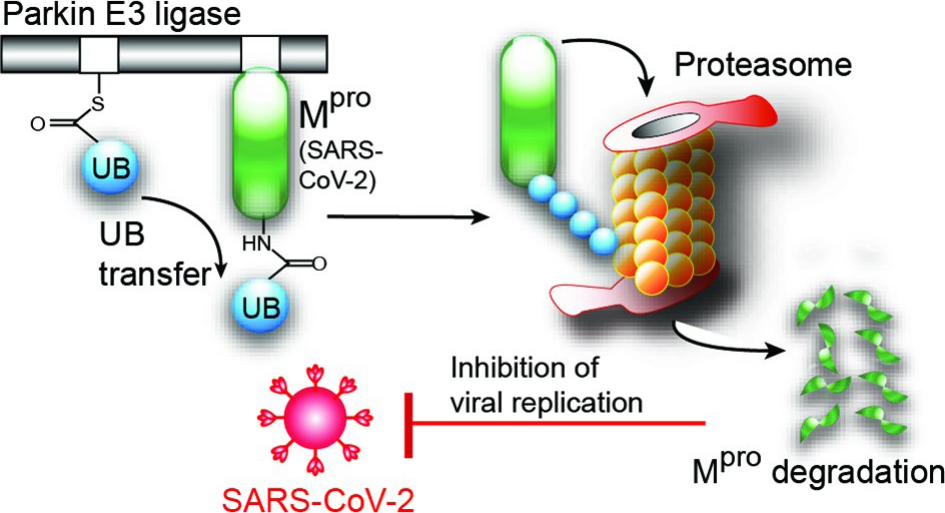

The highly infectious coronavirus SARS-CoV-2 relies on the viral main protease (M^pro^, also known as 3CLpro or Nsp5) to proteolytically process the polyproteins encoded by the viral genome for the release of functional units in the host cells to initiate viral replication. M^pro^ also interacts with host proteins of the innate immune pathways, such as IRF3 and STAT1, to suppress their activities and facilitate virus survival and proliferation. To identify the host mechanism for regulating M^pro^, we screened various classes of E3 ubiquitin ligases and found that Parkin of the RING-between-RING family can induce the ubiquitination and degradation of M^pro^ in the cell. Furthermore, when the cells undergo mitophagy, the PINK1 kinase activates Parkin and enhances the ubiquitination of M^pro^. We also found that elevated expression of Parkin in the cells significantly decreased the replication of SARS-CoV-2 virus. Interestingly, SARS-CoV-2 infection downregulates Parkin expression in the mouse lung tissues compared to healthy controls. These results suggest an antiviral role of Parkin as a ubiquitin ligase targeting M^pro^ and the potential for exploiting the virus–host interaction mediated by Parkin to treat SARS-CoV-2 infection.

The ongoing coronavirus disease 2019 (COVID-19) has become a global pandemic since the spread of the severe acute respiratory syndrome coronavirus-2 (SARS-CoV-2) in early 2020 and has put the world on high alert due to the high infectivity of the virus and the heavy death toll it has caused. The worldwide outbreak of the pandemic has called for a global effort to develop an effective cure of the disease. So far, vaccines and antibody-based treatments have shielded a significant portion of the human population from the diseases associated with SARS-CoV-2 infection.^1,2^ Drugs against specific viral targets, such as remdesivir targeting the RNA-dependent RNA polymerase (RdRp) and paxlovid against the main protease (M^pro^), have been shown to be effective in treating COVID-19 patients.^3,4^ Still, the surge of breakthrough infections by variants such as Delta and Omicron has imposed new risks on vaccinated populations, and strains resistant to antiviral drugs are constantly emerging.^5,6^ Furthermore, the long-persisting disease symptoms of COVID-19 (long COVID) still lack effective treatment and have increased the death rate of patients with chronic commodities.^7^ Understanding the mechanism of virus infection and host response holds the key to developing antiviral drugs and therapies against SARS-CoV-2 infection and future pandemics incited by viral pathogens.

Each step of viral infection, including virion entry, replication, and egress, is a tug-of-war between the virus and the host cells. Protein posttranslational modification (PTM) directly mediate the interactions between SARS-CoV-2 and the host.^8^ On the one hand, viral proteins modify the host proteins to disarm the antiviral defense and promote viral replication. This is manifested by the two proteases, main protease (M^pro^, also known as 3CL^pro^ or Nsp5) and papain-like protease (PL^pro^) encoded by the SARS-CoV-2 genome, that cleave and deactivate host proteins of the innate immune systems.^9−11^ It has been shown that M^pro^ cleaves NEMO and RNF20, and PL^pro^ cleaves the ubiquitin (UB) and ISG15 conjugates in the infected cells to downregulate the expression of type-I interferon (IFN) and dampen the antiviral response of the host.^11−13^ On the other hand, upon detecting viral invasion by the host innate immune sensors such as the pattern-recognition receptors (PRR), host enzymes install a broad spectrum of PTMs on the host and viral targets to decommission them from the viral agenda. For example, the glycosylation of angiotensin-converting enzyme 2 (ACE2) on the host cell surface can block the binding of viral spike protein and viral entry into the host cells.^14^ Acetylation of the viral N protein decreases its affinity with the viral RNA and interferes with virion assembly.^15^ Also, ubiquitination of the spike protein may induce its degradation, and conjugation of ISG15 to the viral proteins inhibits replication and the life cycle of the virus.^16,17^ In this study, we found that the host E3 UB ligase Parkin can ubiquitinate M^pro^ of SARS-CoV-2 and induce its degradation. Furthermore, enhanced Parkin expression in the cell inhibits SARS-CoV-2 replication, and lung tissues of mice infected with SARS-CoV-2 have reduced expression of Parkin. Our study suggests a potential role of Parkin in the host–virus interaction and antiviral response to SARS-CoV-2 based on its activity in ubiquitinating essential viral proteins.

Parkin is an E3 UB ligase in the cytosol, and it has a C-terminal RING-between-RING (RBR) domain equipped with a catalytic Cys residue to uptake UB from the E1 UB activating enzyme and E2 UB conjugating enzyme and pass it on to the substrate proteins.^18−20^ Parkin plays an essential role in the regulation of mitophagy by pairing with the PINK1 kinase to form a feedforward cycle to drive mitophagy initiation.^18,21^ In such a cycle, PINK1 phosphorylates UB attached to the proteins on the surface of damaged mitochondria to recruit Parkin from the cytosol. PINK1 would then phosphorylate Parkin to activate its UB transfer capacity, which would add more UB to the damaged mitochondria. The newly added UB would again be phosphorylated by PINK1 to recruit and activate more Parkin. The propagation of such a cycle quickly decorates the damaged mitochondria with UB chains to facilitate the assembly of autophagosomes. Our work suggests that PINK1 can activate Parkin-mediated ubiquitination of M^pro^ of SARS-CoV-2 and implies a potential crosstalk between the mitophagy regulatory pathways and antiviral response in the host cells.

M^pro^, also known as 3-chymotrypsin-like protease (3CL^pro^) or nonstructural protein 5 (Nsp5), is a crucial target for the development of therapeutics against SARS-CoV-2.^10,22^ Once M^pro^ is self-cleaved from the polyprotein encoded by the Orf1 of the SARS-CoV-1 genome, it can process the polyprotein by cleaving at 11 conserved sites to generate other nonstructural proteins and initiate the viral life cycle in the host cell.^23^ Intense effort on drugging M^pro^ has yielded several inhibitors, including paxlovid, containing the M^pro^ inhibitor nirmatrelvir, as well as ensitrelvir, azuvidine, and deremidevir that have been approved for clinal use in the US or other countries.^24−27^ During the surge of the Omicron variants in 2022, paxlovid has been used to treat SARS-CoV-2 infections and has been proven effective in alleviating the severe symptoms of the disease.^28^ Still, mutations in M^pro^ in SARS-CoV-2 variants have been identified, and they would confer resistance to nirmatrelvir and ensitrelvir.^29,30^ Knowledge of virus–host interaction through M^pro^ regulation by the host enzymes would guide the development of effective treatment of SARS-CoV-2 to counteract its rapid evolution.

## Results

### Parkin-Catalyzed M^pro^ Ubiquitination Is Enhanced by PINK1 in Reconstituted Reactions

**Figure 1:**
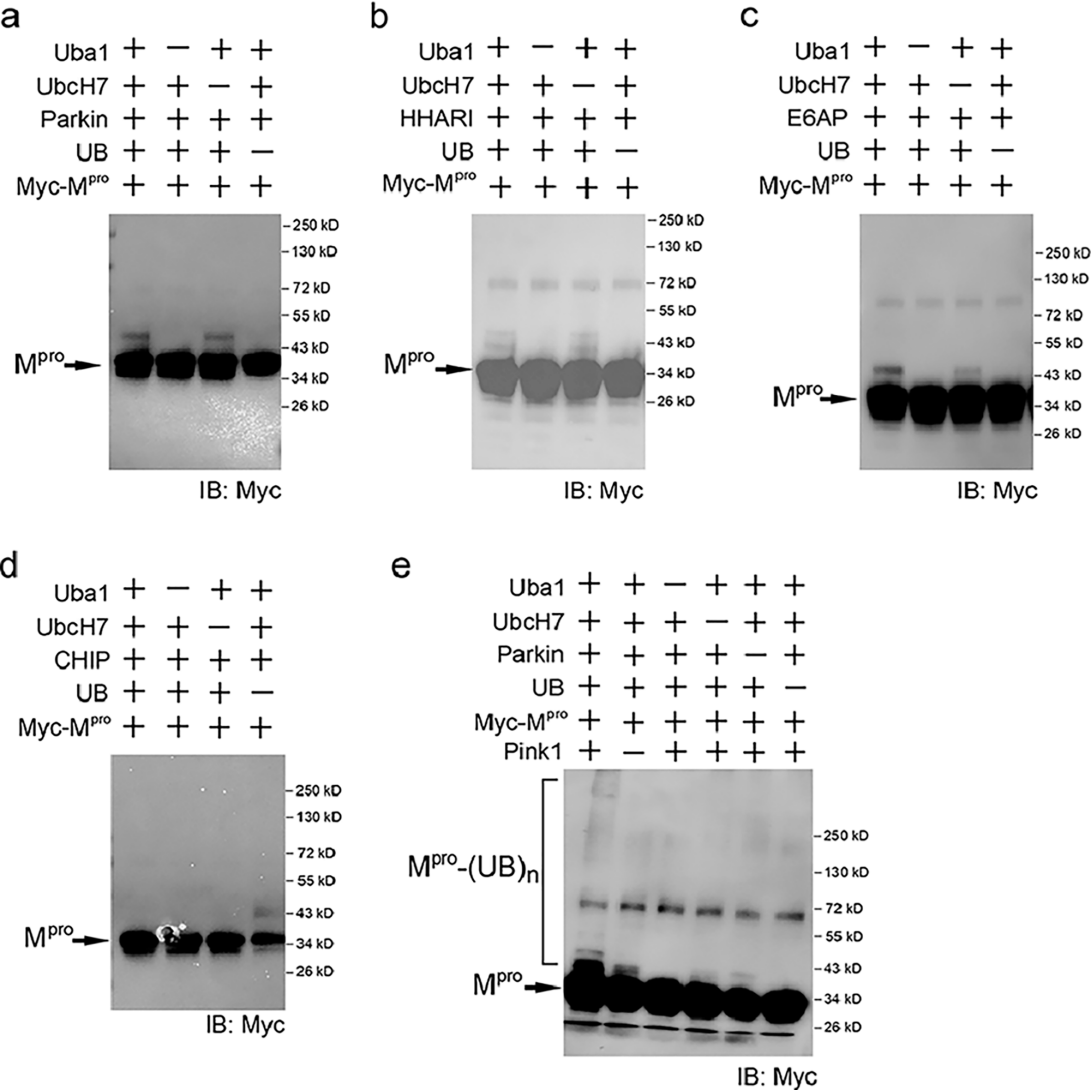
Assaying M^pro^ ubiquitination by various types of E3 UB ligases. M^pro^ of SARS-CoV-2 was expressed from *E. coli* with an N-terminal myc tag, and its ubiquitination was assayed in reconstituted reactions with UB, E1 (Uba1), E2 (UbcH7 or UbcH5b), and various classes of E3 enzymes such as (a) HECT E3 E6AP, (b) RBR E3 Parkin, (c) RBR E3 HHARI, and (d) U-box E3 CHIP. M^pro^ and UB conjugated M^pro^ were detected on the Western blot of the reaction mixture with an anti-myc antibody. Control reactions were set up with the exclusion of E1, E2, and E3 to confirm the dependence of M^pro^ ubiquitination on the UB-transfer cascade. (e) PINK1 enhanced the ubiquitination of M^pro^ in a reconstituted reaction with Parkin and the other components of the UB transfer cascade. In lane 3 of the ubiquitination reactions catalyzed by Parkin (a), HHARI (b), and E6AP (c), bands corresponding to a mono UB -conjugated M^pro^ was observed, suggesting an E2-independent UB transfer from the various E3s to M^pro^. In reconstituted reactions, UB conjugated to Uba1 (E1) can be directly transferred to the HECT (E6AP) and RBR (Parkin and HHARI) E3s and then to the substrates. These results match with previous observations on E2-independent ubiquitination of substrates catalyzed by Parkin and E6AP.^63,64^

It was previously reported that host E3 E6AP/UBE3A catalyzes the ubiquitination of 3C protease of the encephalomyocarditis virus and decreases its presence in infected cells.^31^ This finding prompted us to assay the ubiquitination of M^pro^ of SARS-CoV-2 by E6AP, a HECT-type E3, and other E3 types, such as the RBR E3 Parkin and HHARI and U-box E3 CHIP.^20,32,33^ We set up in vitro ubiquitination reaction of N-terminally tagged Myc-M^pro^ expressed from *E. coli* with purified enzymes of the UB transfer cascades, including E1 (Uba1), E2 (UbcH7 or UbcH5b), and E3s. The addition of UB to the reaction mixture initiated the transfer reaction. Ubiquitination of myc-M^pro^ was assayed by Western blot probed with an anti-myc antibody. We found the ubiquitination reactions with E6AP, Parkin, and HHARI all gave bands corresponding to the mono ubiquitination of M^pro^ (Figure 1a–c). In contrast, CHIP E3 did not generate any ubiquitinated M^pro^ (Figure 1d). Since PINK1 can phosphorylate Parkin to stimulate its UB ligase activity,^34^ we set up in vitro ubiquitination of M^pro^ by Parkin with and without the addition of PINK1. We found that PINK1 enhanced the level of monoubiquitinated M^pro^ species and promoted the formation of polyubiquitinated M^pro^ of higher molecular weight (Figure 1e). These results suggest that M^pro^ is recognized as a ubiquitination target of various E3s, such as E6AP, Parkin, and HHARI, and PINK1 activation of Parkin enhances M^pro^ ubiquitination by Parkin. Since the UB we used in the reconstituted reaction had an HA tag appended to its N-terminus, we also used an anti-HA antibody to detect the polyubiquitination of M^pro^ and the E3 enzymes in the reconstituted reactions and showed the results in Figure S1.

### Parkin Expression Inhibited the Replication of SARS-CoV-2 in HEK293-ACE2 Cells

**Figure 2:**
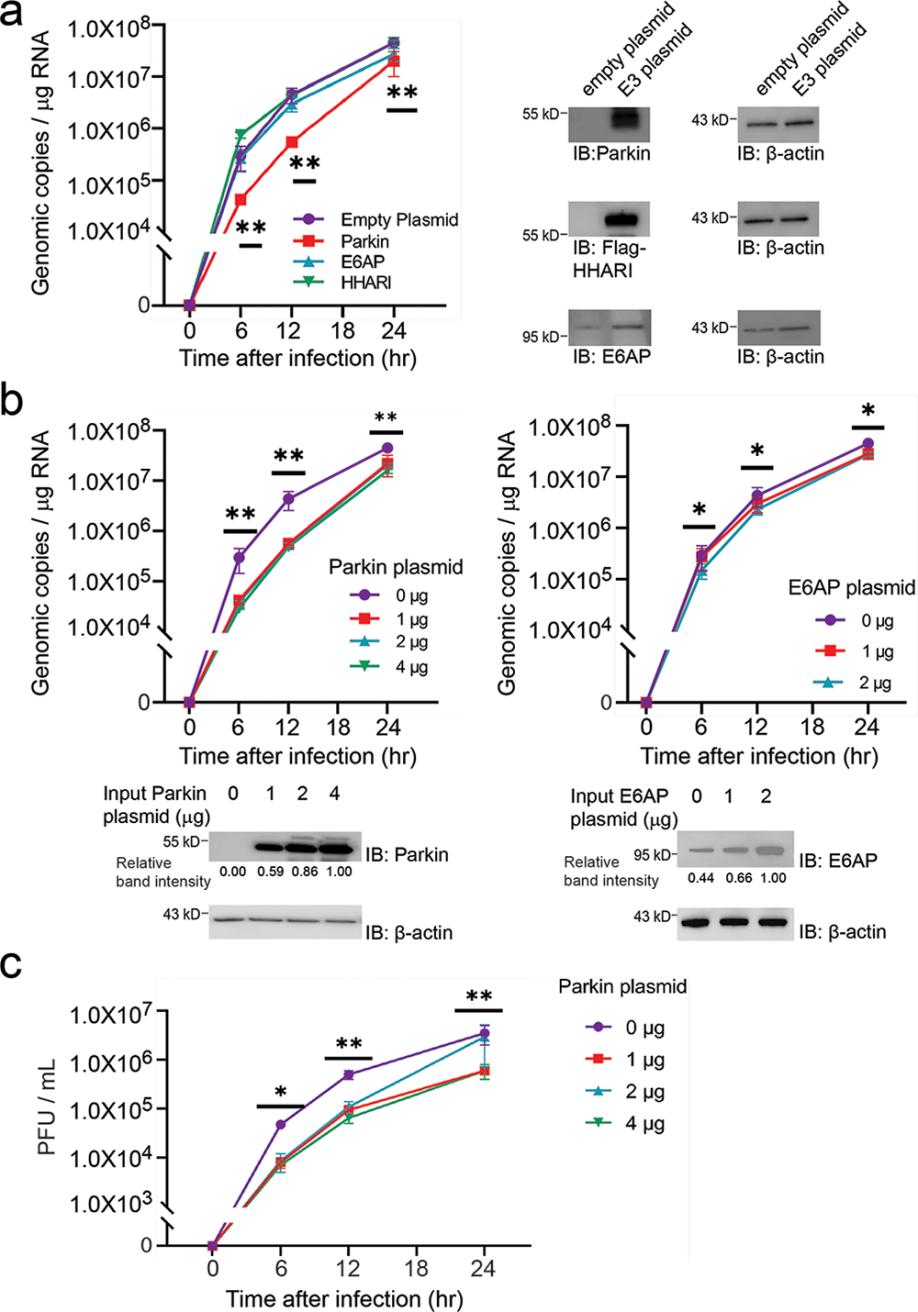
Expression of Parkin in HEK293-ACE2 cells inhibits the replication of SARS-CoV-2. (a) HEK293-ACE2 cells were transfected with 1 μg expression plasmids of Parkin, HHARI, and E6AP. Cells expressing Parkin showed a significant decrease in SARS-CoV-2 replication based on the measurement of viral RNA levels in cell lysates by quantitative RT-PCR. In this comparison, uninfected vs HHARI, showing no significance; uninfected vs Parkin, ** *p* < 0.01; uninfected vs E6AP, *p* < 0.05. (b) HEK293-ACE2 cells were transfected with different amounts of the expression plasmids of Parkin and E6AP. Parkin expression by transfecting the cells with 1–4 μg plasmid can inhibit SARS-CoV-2 replication. In contrast, transfection with an increasing amount of E6AP expression plasmid does not affect the replication of SARS-CoV-2. The relative intensities of Parkin and E6AP bands on the Western blots were quantified by the ImageJ software and shown underneath the blots. Data points show mean ± SE of three experiments. Differences of *p* < 0.05 were considered significant. * *p* < 0.05; ** *p* < 0.01; (c) SARS-CoV-2 titers in culture supernatant were determined by plaque assay using Vero cells. Viral titers are expressed as plaque forming units (PFU)/mL of supernatant. Data points show mean ± SE of three experiments. * *p* < 0.05.

After verifying the recognition of M^pro^ as the substrate of E6AP, Parkin and HHARI in vitro, we were interested in assaying the effects of these E3s on SARS-CoV-2 replication in the cell. We transfected 1 μg expression plasmids of the three E3s into HEK293-ACE2 cells with stable expression of ACE2 that would engage the spike protein of the virus for their infection of the cell. After transfection and verification of the expression of the E3s in HEK293-ACE2 cells, we infected the cells with SARS-CoV-2 and assayed the viral RNA levels in cell lysates at 0, 6, 12, and 24 h after the infection. Based on the measurement of the viral RNA, we found the expression of E6AP and HHARI in the cell has little effect on the virus replication compared to the controls with the transfection of an empty vector (Figure 2a). However, the expression of Parkin in the cells significantly reduced the viral RNA levels at 6 and 12 h after the infection, suggesting Parkin inhibits viral replication in the cell. We also varied the amount of the Parkin plasmid used for transfection to change its expression level in the cell. We found transfection with 1 μg of Parkin plasmid is sufficient to manifest the inhibitory effect of the E3 on virus replication and increasing the amount of Parkin plasmid to 2 or 4 μg for transfection did not give a more potent inhibitory effect (Figure 2b). To evaluate the viral infectivity titers, we performed a plaque assay using cell culture supernatants. Similar to the qRT-PCR results, overexpression of Parkin significantly reduced infectious virus levels in cell supernatant at 6 and 12 h after the infection (Figure 2c). To examine the effects of transfection on cellular cytotoxicity, we utilized an MTT assay to measure cell viability in the SARS-CoV-2-infected and uninfected cells (Figure S2). HEK293-ACE2 cells were transfected with either 1 μg or 4 μg of either E6AP or Parkin expression plasmids. After transfection, we infected the cells with media (uninfected) or SARS-CoV-2 and assayed the cell viability at 24 h. No statistical difference was observed in cell viability after Parkin or E6AP transfection in uninfected cells. Cells without transfection or transfected with 1 μg E6AP plasmid showed a significant decrease in cell survival after being infected with SARS-CoV-2. However, SARS-CoV-2 infection had little effect on the cell viability in cells transfected with 1 or 4 μg of Parkin expression plasmid (Figure S2). These results suggest that Parkin expression has minimal effects on the viability of the cell infected by SARS-CoV-2 and reveal a novel role of Parkin in restricting SARS-CoV-2 replication. Such finding prompted us to verify Parkin-mediated ubiquitination of M^pro^ and the regulation of its stability in the cell.

### Parkin-Induced M^pro^ Ubiquitination and Degradation in the Cell and Enhancement of M^pro^ Ubiquitination with Mitophagy Induction

**Figure 3:**
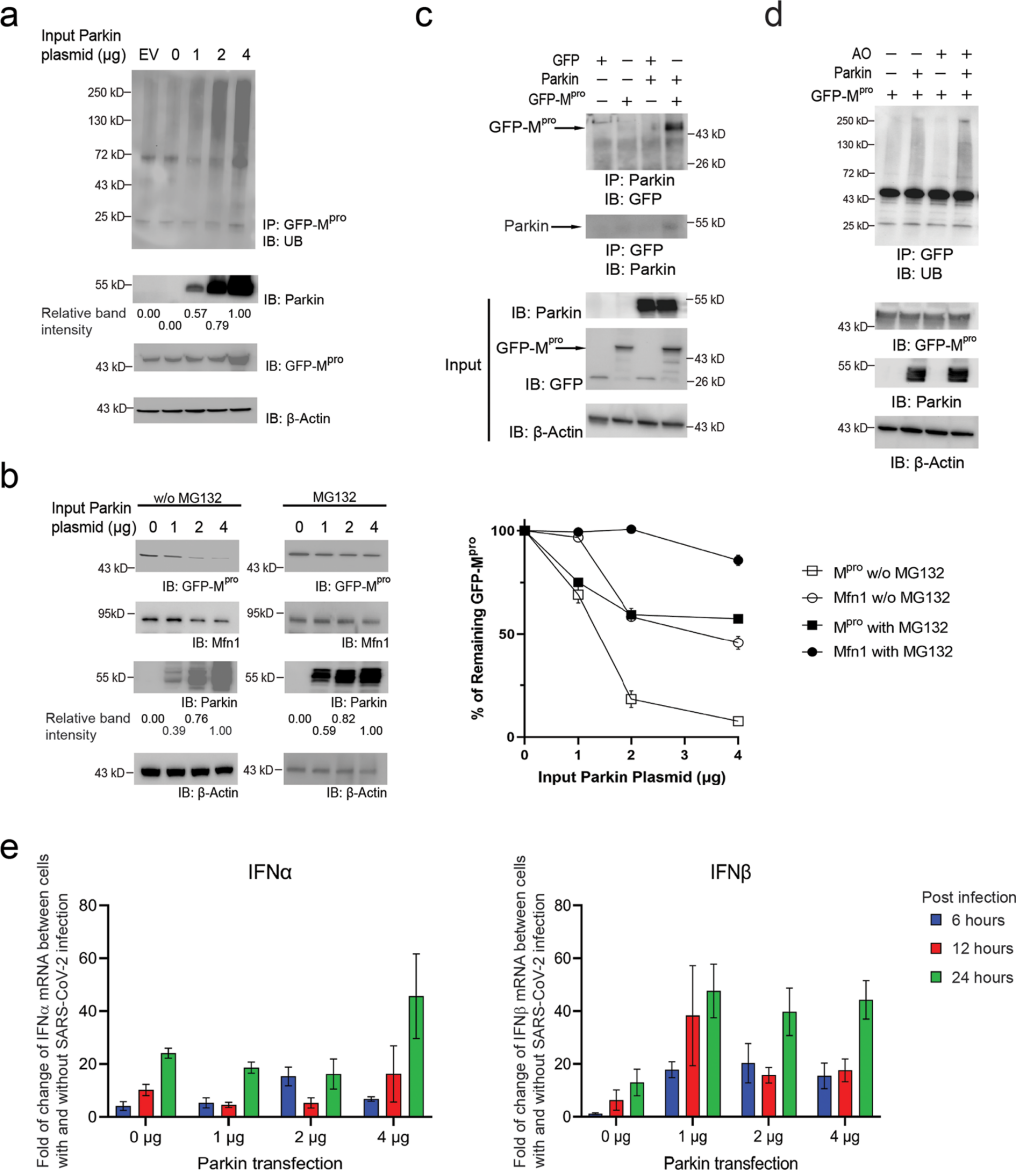
Parkin-induced ubiquitination and degradation of M^pro^ and their interactions in HEK293 cells. (a) Enhanced Parkin expression in the cell correlated with enhanced ubiquitination of M^pro^ with an N-terminal GFP tag. Cells were cotransfected with a fixed amount of the pEBG1-GFP-M^pro^ plasmid and an increasing amount of pLenti-Parkin plasmid and treated with the proteasome inhibitor MG132 4 h before being harvested to inhibit the degradation of ubiquitinated proteins. EV designates the empty vector of the pLenti plasmid used for Parkin overexpression. GFP-M^pro^ was immunoprecipitated from the cell lysate with an anti-GFP antibody, and its ubiquitination level was assayed by Western blot probed with an anti-UB antibody. (b) Parkin expression leads to the destabilization of M^pro^ in the cell. HEK293 cells were transfected with an increasing amount of pLenti-Parkin plasmid with a fixed amount of pEBG-GFP-M^pro^ plasmid. Cells were cultured without MG132 to allow proteasome-mediated degradation of ubiquitinated proteins in the cell. In the control set, 0.5 μM MG132 was added to the cell culture to allow 4 h incubation with the cell to inhibit the proteasome activity. The cell lysates were prepared from both sets and the levels of GFP-M^pro^ in the cell lysates were assayed with an anti-GFP antibody and plotted in the chart together with the levels of Mfn1, a known Parkin substrate. In (a) and (b), the relative intensities of Parkin bands were quantified by the ImageJ software and listed underneath the Western blots probed with an anti-Parkin antibody. For plotting the chart in (b) to show the decreasing levels of GFP-M^pro^ and Mfn1 with increasing amount of Parkin plasmid used for transfection, data points of GFP-M^pro^ and Mfn1 were mean ± SE of three independent experiments with the vertical bars showing SEM of the experiments (*n* = 3). (c) Immunoprecipitation of Parkin or GFP-M^pro^ from the lysates of HEK293 cells expressing the two proteins. Immunoprecipitation of Parkin can pull down GFP-M^pro^ but not GFP by itself, and immunoprecipitation of GFP-M^pro^ can pulldown Parkin, suggesting the interaction between Parkin and GFP-M^pro^ in the cell. (d) Induction of mitophagy by treating the cells with a combination of antimycin and oligomycin (AO) enhanced the ubiquitination of GFP-M^pro^. Cells with and without the overexpression of Parkin were treated with AO and the proteasome inhibitor MG132 to accumulate ubiquitinated species. GFP-M^pro^ was then immunoprecipitated from the cell lysate with an anti-GFP antibody, and its ubiquitination was probed with an anti-UB antibody. (e) Fold of change of mRNA levels of IFNα and IFNβ in cells infected with SARS-CoV-2 relative to uninfected cells. HEK293-ACE2 cells were transfected with an increasing amount of Parkin expression plasmids (0–4 μg) and infected by SARS-CoV-2 for 6, 12, and 24 h. The cells were lysed for assaying IFNα and IFNβ levels by qRT-PCR. In the control set, HEK293-ACE2 cells were transfected with the same amount of Parkin expression plasmids without virus infection. The ratio of IFNα and IFNβ mRNA levels in virus infected and uninfected cells were calculated to reveal the IFN response to SARS-CoV-2 infection.

To check the dependence of M^pro^ ubiquitination on Parkin, we coexpressed GFP-M^pro^ with an increasing amount of Parkin in HEK293 cells. We treated the cells with the proteasome inhibitor MG132 for 4 h before harvesting the cells to accumulate ubiquitinated species. GFP-M^pro^ was immunoprecipitated from the cell lysate with an anti-GFP antibody, and the ubiquitination level of the protein was assayed by Western blotting probed with an anti-UB antibody (Figure 3a). We found an increased expression of Parkin resulted in a corresponding increase in the ubiquitination of M^pro^, suggesting that Parkin recognizes M^pro^ as the substrate for the ubiquitination reaction in the cell.

To check if Parkin-mediated M^pro^ ubiquitination would destabilize M^pro^ and decrease its presence in the cell, we again coexpressed GFP-M^pro^ with an increasing amount of Parkin in HEK293 cells, but this time, we did not treat the cell with MG132 before harvesting the cells. We found that the level of GFP-M^pro^ decreased with the increasing amount of Parkin expression, and the level of Mfn1, a known Parkin substrate in the cell,^35^ also decreased with increasing Parkin expression (Figure 3b). In the control set where MG132 was added to the cell culture to inhibit proteasome activity, both GFP-M^pro^ and Mfn1 were maintained at a higher level compared to the set without MG132 added. These results confirm that Parkin regulates the stability of M^pro^ in the cell by inducing its ubiquitination and degradation by the proteasome.

In another experiment, we expressed Parkin with either GFP-M^pro^ fusion or the GFP tag only in HEK293 cells and carried out immunoprecipitation of the cell lysates with an anti-Parkin or an anti-GFP antibody. We found the anti-Parkin antibody could coimmunoprecipitate GFP-M^pro^ but not GFP itself, and the anti-GFP antibody could coimmunoprecipitate Parkin (Figure 3c). These results proved that Parkin and M^pro^ can directly interact with each other to enable Parkin-catalyzed ubiquitination of M^pro^.

Since the in vitro assay showed that PINK1 can stimulate the ubiquitination of M^pro^ by Parkin (Figure 1e), we assayed if the activation of Parkin by PINK1 during mitophagy induction could enhance M^pro^ ubiquitination in the cell. We expressed GFP-M^pro^ in HEK293 cells alone or with the coexpression of Parkin. We then treated cells with a combination of antimycin and oligomycin (AO) that would induce mitochondrial depolarization and recruit PINK1 and Parkin to the damaged mitochondria.^36^ PINK1 would then phosphorylate Parkin and stimulate its ubiquitination of substrate proteins. Indeed, GFP-M^pro^ showed enhanced ubiquitination in cells treated with AO compared to cells without AO treatment (Figure 3d). Also, the combination of Parkin - GFP-M^pro^ coexpression and AO treatment of the cells gave the highest level of M^pro^ ubiquitination. These results suggest the PINK1-Parkin forward cycle formed in response to mitochondria stress would accelerate the ubiquitination of M^pro^ during the viral infection.

M^pro^ has been shown to restrict type-1 interferon expression.^37^ The ubiquitination and subsequent degradation of this protease could potentially upregulate type-1 interferon (IFN) response. Therefore, we measured transcript levels of IFNα and IFNβ in Parkin expressing cells after SARS-CoV-2 infection. SARS-CoV-2 infection results in a modest upregulation in the RNA levels of IFNα and IFNβ in the cells transfected with an empty vector. Interestingly, we observed a significantly high expression of IFNα at 24 h in Parkin-expressing cells. Similarly, IFNβ shows a significant upregulation in Parkin-expressing cells in comparison to the empty-vector transfected cells at all time points and transfection amounts (Figure 3e). Previously, IFNγ has been shown to induce the expression of Parkin in airway epithelial cells and elevate the neutrophilic inflammation.^38^ These results suggest a cross-regulation between Parkin and IFN to promote the antiviral response.

### SARS-CoV-2 Infection Decreased the Expression Level of Parkin in the Mouse Lung Tissues

**Figure 4:**
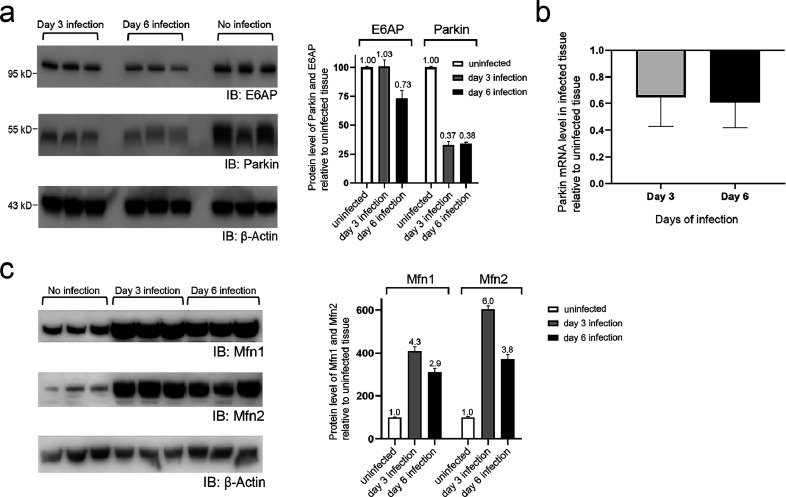
Mouse lung tissues with SARS-CoV-2 infection showed a decreased level of Parkin expression. (a) The protein levels of E3 enzymes Parkin and E6AP in the mouse lung tissue on day 3 (lanes 1–3) or day 6 (lanes 4–6) of SARS-CoV-2 infection was compared with the healthy lung tissue by Western blotting probed with an anti-Parkin or an anti-E6AP antibody. The levels of Parkin and E6AP in infected and healthy lung tissues are normalized against the level of β-actin and plotted in the bar charts. Data points show mean ± SE of three or more experiments, and the ratios of E6AP and Parkin levels in infected and mock-infected lung tissues are displayed above the bars. (b) Fold of change of mRNA levels of Parkin in mouse lung tissues infected with SARS-CoV-2 relative to uninfected tissues. (c) Levels of Mfn1 and Mfn2 in mouse lung tissues with no infection of SARS-CoV-2 or on day 3 or 6 of infection with the virus. The levels of Mfn1 and Mfn2 in infected and healthy lung tissues are normalized against the level of β-actin and plotted in the bar charts. Data points show mean ± SE of three or more experiments, and the ratios of protein levels in infected and mock-infected lung tissues are displayed above the bars.

We also checked the effects of SARS-CoV-2 infection on Parkin expression in mouse lung tissues. We collected the lung tissues of mock (PBS)- and SARS-CoV-2-infected mice on day 3 or 6 after the infection, prepared the tissue lysates, and assayed the levels of Parkin by Western blotting probed with an anti-Parkin antibody. We found that the Parkin level in lung tissues of mice with SARS-CoV-2 infection was less than 40% of the Parkin level in the control mice on days 3 and 6 after virus infection (Figure 4a). In comparison, another E3, E6AP, that does not affect SARS-CoV-2 replication, showed similar levels in the lung tissues of the infected and control mice on day 3 of the infection and showed a small decrease (∼25%) in the infected lung tissues on day 6 of the infection (Figure 4a). We also performed RT-PCR with Parkin-specific probes and found the mRNA level of Parkin was reduced in lung tissues with SARS-CoV-2 infection by averages of 35 and 40% compared to healthy lung tissues (Figure 4b). These results suggest that the SARS-CoV-2 virus may downregulate Parkin expression in the lung tissues to promote viral replication. To probe the effect of SARS-CoV-2 infection on the mitochondrial proteins, we analyzed the levels of mitochondrial outer membrane proteins Mfn1 and Mfn2 in the mouse lung tissue without virus infection and on 3 or 6 days after viral infection. We found that there was a significant increase of Mfn1 and Mfn2 levels in the infected lung tissues compared to tissues without the infection by SARS-CoV-2 (Figure 4c). Since Mfn1 and Mfn2 are common mitochondrial markers,^35,39^ our results suggest SARS-CoV-2 infection may affect mitochondrial contents and their activities in the cell. The change of mitophagy activity in SARS-CoV-2 infected cells and tissues and the mechanistic linkage between viral infection and mitophagy response warrant further investigation.

## Discussion

Our work identified M^pro^, an essential protease for SARS-CoV-2 replication and virus-host interaction, as a substrate of the host E3 Parkin, and Parkin-catalyzed M^pro^ ubiquitination signals its degradation in the cell. M^pro^ plays an important role in the viral lifecycle by cleaving polyproteins encoded by the SARS-CoV-2 genome for releasing functional viral proteins to assemble the replication and transcription complexes in the infected cells.^23^ In addition, M^pro^ interacts with the innate immune system to compromise its activity and promote viral replication. The PRR sensors in the host cells detect pathogen-associated molecular patterns (PAMP), such as the RNA fragments of the SARS-CoV-2 genome. Following PRR activation, interferon regulatory factors 3 and 7 (IRF3 and IRF7) are phosphorylated and transported into the nucleus to induce the expression of interferons (IFNs) and proinflammatory cytokines and chemokines.^40,41^ M^pro^ has been found to affect the nuclear transport of phosphorylated IRF3 and suppress the production of IFN and cytokines to attenuate the host inflammatory response.^42^ M^pro^ also interacts with STAT1 and induces its degradation by autophagy to suppress IFN production regulated by the JAK-STAT signaling pathway.^37^ So, Parkin-catalyzed ubiquitination of M^pro^ represents a defensive PTM in the host cells to fend off SARS-CoV-2 infection by eliminating this essential viral protease.

It was recently found that Ring E3 UB ligase TRIM7 recognizes a C-terminal degron motif of substrate proteins that folds into an α-helix ending with a hydrophobic residue and a Gln residue at the C-terminus.^43,44^ The C-terminal Gln motif can be generated by M^pro^ cleavage of viral and host proteins, and M^pro^ itself also has the degron motif due to its self-cleavage from the polyprotein. Indeed, the ubiquitination of M^pro^ by TRIM7 was confirmed, and TRIM7 was found to destabilize M^pro^ in the cell. However, the expression of TRIM7 does not restrict the replication of SARS-CoV-2, although it can inhibit the replication of Coxsackie virus and norovirus.^44^ In contrast, our study confirms that Parkin expression has a significant effect on SARS-CoV-2 replication, suggesting a unique role of Parkin in the host antiviral response against SARS-CoV-2. The interaction motif in M^pro^ for Parkin recognition warrants elucidation to reveal how host E3s differ in ubiquitinating the viral proteins and regulating their activities in the cell.

Parkin’s newly discovered activity in M^pro^ ubiquitination may be integrated into a large program implemented by the host ubiquitin-proteasome system (UPS) to respond to SARS-CoV-2 infection. So far, a few protein ubiquitination pathways have been identified to counteract the invasion by SARS-CoV-2. For example, Ring E3 ligase TRIM13 and RNF121 catalyze the polyubiquitination of SARS-CoV-2 proteins Nsp6 and ORF7a, respectively.^45^ The polyubiquitinated viral proteins then recruit the IKK kinase complex and NEMO to activate the antiviral response signaled by NF-κB. Another E3 RNF5 has been found to catalyze the ubiquitination of the E protein of SARS-CoV-2 that would lead to the degradation of E by the UPS and inhibition of virus replication.^46^ MARCH8 E3 ubiquitinates the viral spike protein and induces its degradation to manifest its antiviral function.^47^ To escape regulation by the host UPS, SARS-CoV-2 has developed capacities to diverge the targeting of host E3s. ORF10 of the virus can bind to Cul2-ZYG11B to stimulate the activity of the E3 complex in ubiquitinating and degrading ciliary proteins that lead to the loss of cilia decorating the cell surface.^48^ M protein of the virus is ubiquitinated by RNF5 to strengthen its binding with the viral E protein and facilitate the assembly and egress of the virion particle.^49^ Ubiquitination of Orf7a of SARS-CoV-2 inhibits IFN signaling and reduces ER stress and apoptosis of the infected cell to promote viral replication.^50,51^ Overall, UPS may play a multifaceted role in the viral-host interaction during SARS-CoV-2 infection. A comprehensive survey of the UPS system has identified numerous E3 UB ligases or deubiquitinating enzymes (DUBs) that can either enhance or suppress the replication of SARS-CoV-2.^52^ It would be interesting to further characterize the role of Parkin in collaborating with or acting against other E3s to defend the host cells from viral infection.

In this study, we found that overexpression of Parkin in HEK293-ACE2 cells infected with SARS-CoV-2 reduced infectious viral titers (Figure 2). We also revealed that Parkin ubiquitinates M^pro^ and signals its degradation. Furthermore, when the cells undergo mitophagy upon AO treatment, Parkin activation by PINK1 enhances M^pro^ ubiquitination. These results suggest mitophagy in the cell would lead to enhanced ubiquitination of M^pro^ due to the action of the PINK1-Parkin forward feeding cycle, so mitophagy induction and Parkin activation may be a part of the antiviral response of the host cell. In addition, we show that Parkin expression accompanied by ubiquitination of M^pro^ results in upregulation of type-1 interferon response, suggesting a potential mechanism of Parkin-mediated restriction of SARS-CoV-2 replication (Figure 3e). It was recently reported that cells infected by SARS-CoV-2 showed an accumulation of Parkin and PINK1 on mitochondria to initiate mitophagy, but the mitophagy process was stopped at the stage of autophagosome formation.^53^ Mitophagy inhibition was also identified in cells infected by other viruses. For example, HCV infection inhibits mitophagy by blocking Parkin deposition to the damaged mitochondria.^54^ Cells infected by dengue virus showed mitochondrial stress, but mitophagy was inhibited due to virus-induced downregulation of PINK1 and Parkin expression.^55^ Matching with these studies, we found Parkin expression in mouse lung tissues infected with SARS-CoV-2 was reduced by more than 60% compared with healthy lung tissues (Figure 4a). These results suggest that the virus may develop mechanisms to inhibit mitophagy and suppress the antiviral activity of Parkin.

Reduced Parkin levels in lung tissues infected by SARS-CoV-2 may lead to the injury of lung tissues due to the suppressed activity of Parkin and mitophagy. In light of this, restoring mitophagy may be a useful direction for the development of therapeutics for COVID-19. Still, Parkin was found to have a mixed role in the virus-host interaction. A few studies suggest that Parkin targets key components of the antiviral response pathways, such as RIG-I, MDA5, and TRAF3, for ubiquitination and degradation, resulting in the delay of IFN activation.^56,57^ So Parkin is exploited by classical swine fever virus (CSFV), coxsackievirus B3 (CVB3), and hepatitis B virus (HBV) to attenuate the immune response.^58−60^ It warrants further study on the unique role of Parkin on SARS-CoV-2 infection. Furthermore, there are cases suggesting a causative role of SARS-CoV-2 infection in the development of Parkinson’s disease (PD) that are genetically related to the mutations in Parkin.^61,62^ Our study showing suppressed expression of Parkin by SARS-CoV-2 may provide a mechanistic linkage between PD and COVID-19.

## Methods

### Plasmids and Reagents

XL1 Blue cells were from Agilent Technologies (Santa Clara, CA, USA). BL21 (DE3) pLysS chemical competent cells were from Invitrogen. pET-15b and pET-28a plasmids for protein expression were from Novagen (Madison, WI, USA). pET plasmids were constructed for protein expression, including pET28a-Uba1, pET15b-UbcH7, pET15b-UbcH5b, pET28a-E6AP, pGEX-4T-1-Parkin, pGEX-4T-1-HHARI, pET15b-UB, and pET28a-M^pro^. PINK1 was purchased from Boston Biochem (AP-182–100). HEK293T cells were from American Tissue Culture Collection (ATCC) and cultured in high-glucose Dulbecco’s modified Eagles medium (DMEM) (Life Technologies, Carlsbad, CA, USA) with 10% (v/v) fetal bovine serum (FBS) (Life Technologies). The anti-UB antibody (sc-8017), anti-Myc antibody (sc-40), anti-HA antibody (sc-7392), anti-GFP antibody (sc-9996), anti-E6AP antibody (sc-25509), anti-Parkin antibody (sc-32282), and anti-β-actin antibody (sc-47778) were from Santa Cruz Biotechnology. The anti-Flag M2 antibody (F3165) was from Sigma-Aldrich. The anti-Mfn1 antibody (A9880), anti-Mfn2 antibody (A12771), and Rabbit anti-GFP antibody (AE078) were from ABclonal Technology. The antibodies were diluted between 500- and 1000-fold to probe the Western blots.

### Protein Ubiquitination Assays

All assays were set up in a 50 μL reaction in buffer containing 50 mM Tris, 5 mM MgCl_2_, 5 mM ATP, and 1 mM DTT. In the reconstituted reaction, 5 μM Myc-tagged M^pro^ was incubated with 0.5 μM Uba1, 0.5 μM UbcH7 or UbcH5b, 10 μM UB, and various E3 ligases, including N-terminal Flag-tagged E6AP, Flag-tagged CHIP, GST-tagged Parkin and GST-tagged HHARI, respectively, at 37 °C for 8–10 h. The reactions were then quenched by boiling in the sample loading buffer of SDS-PAGE with DTT for 5 min and analyzed by SDS-PAGE. The protein bands on the PAGE gel were transferred to a Western blot that was probed with an anti-myc antibody. In another experiment, 0.5 μM wt PINK1 was added to the reaction mixture of M^pro^ with Parkin and other supporting components, including Uba1, UbcH7, and UB. The reactions were incubated for 8–10 h at 37 °C and then quenched by boiling in the sample loading buffer with DTT for 5 min and analyzed by SDS-PAGE. The Western blot of the PAGE gel was probed with an anti-myc antibody.

### Cell-Based Assay to Verify M^pro^ Ubiquitination by Parkin with the Induction of Mitophagy

HEK293T cells were transfected with varying amounts of pLenti-Parkin plasmid at 0, 1, 2, and 4 μg and 1 μg pEBG-GFP-M^pro^ plasmid for 14 h. The cells were treated with 0.5 μM MG132 for an additional 12 h. Cells were then lysed and washed twice with ice-cold PBS, pH 7.4, followed by the addition of 1 mL ice-cold RIPA buffer that was allowed to incubate with the cells at 4 °C for 10 min. The cells were disrupted by repeated aspiration through a 21-gauge needle to induce cell lysis and the cell lysate was transferred to a 1.5 mL tube. The cell debris in the lysate was pelleted by centrifugation at 13,000 rpm. for 20 min at 4 °C, and the supernatant was transferred to a new tube and precleared by adding 1.0 μg of the appropriate control IgG (normal mouse or rabbit IgG corresponding to the host species of the primary antibody). Twenty μL of suspended Protein A/G PLUS-agarose was added to the supernatant, and the incubation was continued for 30 min at 4 °C. After this, cell lysate containing 2 mg total protein was transferred to a new tube and 30 μL (i.e., 6 μg) primary antibody specific for GFP was added to bind to GFP-M^pro^ in the lysate. The incubation was continued for 1 h at 4 °C, and 30 μL of resuspended protein A/G PLUS-agarose was added. The tubes were capped and incubated at 4 °C on a rocking platform overnight. The next day, the agarose beads were pelleted by centrifugation at 350*g* for 5 min at 4 °C. The beads were then washed three times, each time with 1.0 mL PBS. After the final wash, the beads were resuspended in 40 μL of 1× Laemmli buffer with β-mercaptoethanol. The samples were boiled for 5 min and analyzed by SDS-PAGE. The Western blots of the PAGE gels were probed with an anti-UB antibody. For assaying the effect of Parkin activation on the ubiquitination of the virus M^pro^ during mitophagy, cells were treated with 4 μM antimycin A and 10 μM oligomycin (AO) for 1 h, and GFP-M^pro^ was immunoprecipitated for assaying its ubiquitination level following the procedures above.

### Protein Stability Assays

To examine the effect of Parkin on the steady-state levels of M^pro^, HEK293T cells (5 × 10^6^ cells) were transiently transfected with varying amounts of pLenti plasmid of Parkin with DharmaFECT kb transfection kit. Cells were harvested at 24 h after transfection, and the amount of GFP-M^pro^ proteins in the cell lysate was assayed by immunoblotting with an anti-GFP antibody.

### Coimmunoprecipitation to Detect Parkin-Mpro Interaction

HEK293T cells were cotransfected with pLenti-Parkin and pEBG-GFP-M^pro^ plasmids for 13–16 h. For coimmunoprecipitation, cell lysates were incubated overnight with mouse anti-Parkin or rabbit anti-GFP antibodies at 4 °C, followed by 4 h incubation with protein A/G-Sepharose beads (Santa Cruz Biotechnology). Coprecipitated proteins were analyzed by immunoblotting using rabbit anti-GFP or mouse anti-Parkin antibodies, respectively.

### *In Vitro* SARS-CoV-2 Infection and Plaque Assay

Transformed HEK293-ACE2 cells (BEI# NR-52511) were plated in 12-well plates at 80% confluency and infected with SARS-CoV-2 B.1 Wuhan virus (BEI# NR-52281) at an MOI of 0.1. Briefly, the cells were incubated with SARS-CoV-2 for 1 h at 37 °C. After incubation, the cells were washed with PBS and replenished with fresh DMEM media. Then, supernatant and cell lysates were collected at 0, 6, 12, and 24 h. Viral titers were measured in cell culture supernatants by plaque assay using Vero E6 cells. Viral titration was performed by a 10-fold serial dilution in DMEM and then applied to a confluent monolayer of cells. Cells were subject to a 1 h infection followed by a 1% agarose overlay. Cells were then further incubated for 48 h and then stained with 2% neutral red to visualize plaque formation.

### *In Vivo* Mouse Challenge and Tissue Collection

All mouse experiments were conducted in Animal Biosafety Level 3 in strict accordance with the standard operating procedure. The protocol was approved by the Georgia State University Institutional Animal Care and Use Committee (Protocol number A20044). Hemizygous K18-hACE2 mice (2B6.Cg-Tg (K18-ACE2)2Prlmn/J) were obtained from Jackson Laboratory. Eight-week-old hemizygous K18-hACE2 were intranasally infected with 10,000 PFU of B.1 Wuhan virus (BEI# NR-52281) and euthanized on days 3 and 6 after the infection. Mice were perfused with cold PBS, and the lung tissues were flash-frozen in 2-methylbutane (Sigma, St. Louis, MO, USA). The lung tissues were lysed by RIPA buffer, followed by Western blot analysis, to detect the endogenous levels of E3 enzymes E6AP and Parkin, and mitochondrial proteins Mfn1 and Mfn2.

### RNA Extraction and Quantitative RT-PCR

Total RNA was extracted from cell lysates using a Qiagen RNeasy Mini kit (Qiagen, Germantown, MD, USA). Quantification of viral RNA levels from HEK293-ACE2 cells was performed. RNA extracted from infected cells was quantified and normalized, and total viral RNA per μg of total cellular RNA was calculated. qRT-PCR was then performed to measure RNA levels using previously published protocols, primers, and probes specific to SARS-CoV-2 forward (5′-GACCCCAAAATC AGCGAAAT-3′), reverse (5′-TCTGGTTACTGCCAGTTGAATCTG-3′), probe, (5′-FAM-ACCCCGCATTACGTTTGGTGGACC-BHQ1–3′) targeting the SARS-COV-2 N1 region (Integrated DNA Technologies). Viral RNA copies were then determined by comparison with a standard curve generated from a 10-fold serial dilution of SARS-CoV-2 RNA. Quantification of IFNα and β levels were also determined using cell lysates by qRT-PCR. Following extraction of total RNA, cDNA library was created using a Biorad iScriptTM cDNA synthesis kit. Human-specific primers for IFNα and IFNβ were used in conjunction with Biorad SsoAdvanced Universal SYBR Green Supermix. The fold of change in SARS-CoV-2-infected cells compared to corresponding uninfected cells was calculated after normalizing to the housekeeping GAPDH gene. For measuring the Parkin mRNA level in mouse lung tissues, the total RNA was isolated from lung tissues on days 3 and 6 of SARS-CoV-2 infection using a Qiagen RNeasy Mini kit (Qiagen). cDNA was then generated using an iScriptTM cDNA synthesis kit (Bio-Rad). Then, qRT-PCR was conducted using the cDNAs to determine the expression levels of Parkin. Mouse-specific primers for Parkin (Mm01323528_m1) were obtained from Applied Biosystems.The fold change in SARS-CoV-2-infected tissues compared to mock-infected tissues was calculated after normalizing to the housekeeping GAPDH gene.

### Cell Proliferation Assay

HEK293-ACE2 cells were transfected with either 1 μg or 4 μg of E6AP or Parkin expression plasmid. Twenty four hours after the transfection, cells were infected with media (uninfected) or SARS-CoV-2 at MOI 0.1 for 1 h. Cells were washed twice with PBS and then incubated for an additional 24 h. Cell viability was assessed at 24 h after infection using CellTiter 96 AQueous One Solution proliferation Asssay (Promega).

### Statistical Analysis

To compare the viral RNA copies in the cell lysates and virus infectivity titers with the expression of different E3 ligases, a two-way analysis of variance (ANOVA) with Dunnett’s multiple comparisons test was used to calculate the *p* values. An unpaired Student’s *t* test was used to calculate the *p* values for comparing the changes in viral RNA copies and cell viability by varying the amounts of transfected plasmids of E3 ligases. Differences with *p* values of <0.05 were considered significant.

## Supporting Information Available

(Figure S1) In vitro ubiquitination reactions of M^pro^ protein of SARS-CoV-2 by Parkin, HHARI, E6AP, and CHIP probed with anti-HA antibody to detect UB-conjugates; (Figure S2) MTT assay to measure the effect of E6AP and Parkin plasmid transfection and SARS-CoV-2 infection on cell viability (PDF)

## ABBREVIATIONS

ACE2: angiotensin-converting enzyme 2
CSFV: classical swine fever virus
CVB3: coxsackievirus B3
DUB: deubiquitinating enzyme
HBV: hepatitis B virus
IFN: interferon
IRF3 and IRF7: interferon regulatory factors 3 and 7
M^pro^: main protease
Nsp5: nonstructural protein 5
PAMP: pathogen-associated molecular patterns
PD: Parkinson’s disease
PLpro: papain-like protease
PRR: pattern-recognition receptor
PTM: protein posttranslational modification
RBR: RING-between-RING
RdRp: RNA-dependent RNA polymerase
UPS: ubiquitin-proteasome system
