# Supplementary material for: Ubiquitin Ligase Parkin Regulates the Stability of SARS-CoV-2 Main Protease and Suppresses Viral Replication
Source: ACS Infect Dis. 2024 Feb 22;10(3):879–89. doi: 10.1021/acsinfecdis.3c00418 (PMC10928718; doi:10.1021/acsinfecdis.3c00418)
Supplement: Supplementary file 1 — id3c00418_si_001.pdf [file id3c00418_si_001.pdf]

Supporting Information

Ubiquitin Ligase Parkin Regulates the Stability of SARS-CoV-2 Main Protease and Suppresses Viral Replication

Li Zhou[1, †], Ruochuan Liu[1, †], Heather Pathak[2, †], Xiaoyu Wang[1], Geon H. Jeong[1], Pratima Kumari[2], Mukesh Kumar[2,*], and Jun Yin[1,*]

[1]Department of Chemistry and Center for Diagnostics and Therapeutics, Georgia State University, Atlanta, GA 30303, United States

[2]Department of Biology and Center for Diagnostics and Therapeutics, Georgia State University, Atlanta, GA 30303, United States

[†]These authors made equal contributions to this work.

*To whom correspondence should be addressed: Mukesh Kumar (mkumar8@gsu.edu) and Jun Yin (junyin@gsu.edu)

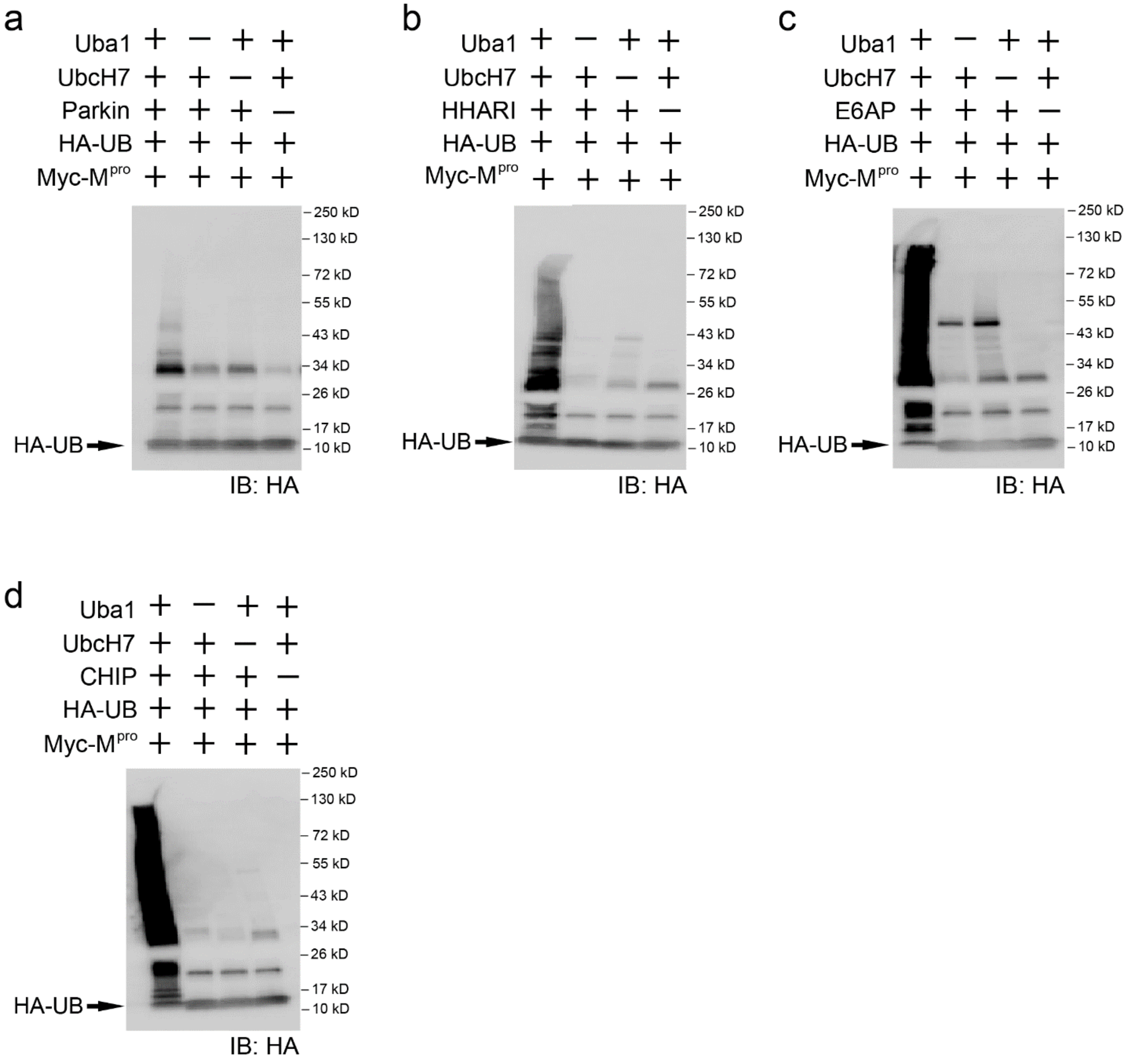

Supporting Figure S1. In vitro ubiquitination reactions of myc-tagged M^{pro} protein of SARS-CoV-2 catalyzed by Parkin (**a**), HHARI (**b**), E6AP (**c**), and CHIP (**d**). The western blots of the reconstituted reactions were probed with an anti-HA antibody to detect ubiquitinated M^{pro} or E3 species that were conjugated with UB fused to an N-terminal HA tag.

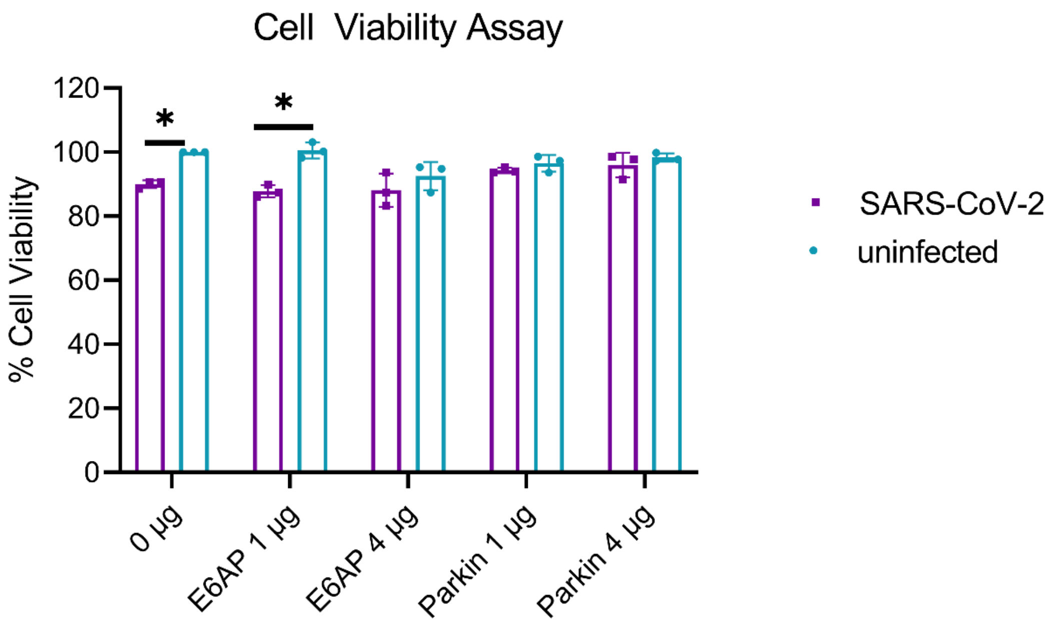

Supporting Figure S2. The viability of the cells transfected with plasmids expressing E6AP or Parkin at 24 hours after SARS-CoV-2 infection. Cell viability was assessed by cell proliferation assay and the percentages of cell viability were calculated by comparing to corresponding uninfected cells. Data are expressed as mean ± SE conducted in triplicate. * $p < 0.05$.
